# Risk of Adverse Maternal Outcomes in Pregnant Women With Disabilities

**DOI:** 10.1001/jamanetworkopen.2021.38414

**Published:** 2021-12-15

**Authors:** Jessica L. Gleason, Jagteshwar Grewal, Zhen Chen, Alison N. Cernich, Katherine L. Grantz

**Affiliations:** 1 Epidemiology Branch, Division of Intramural Population Health Research, *Eunice Kennedy Shriver* National Institute of Child Health and Human Development, National Institutes of Health, Bethesda, Maryland; 2 Office of the Director, *Eunice Kennedy Shriver* National Institute of Child Health and Human Development, National Institutes of Health, Bethesda, Maryland; 3 Biostatistics and Bioinformatics Branch, Division of Intramural Population Health Research, *Eunice Kennedy Shriver* National Institute of Child Health and Human Development, National Institutes of Health, Bethesda, Maryland

## Abstract

**Importance:**

Women with disabilities have a higher risk of preterm birth, gestational diabetes, preeclampsia, and cesarean delivery; however, their risk of other obstetric interventions, adverse maternal outcomes, and clinical indications for increased cesarean delivery is unclear.

**Objective:**

To evaluate risk of a range of obstetric interventions and adverse maternal outcomes, including severe maternal morbidities (SMM) and mortality, among women with and without disabilities.

**Design, Setting, and Participants:**

The Consortium on Safe Labor was a retrospective cohort that included comprehensive medical chart review for deliveries between January 2002 and January 2008. Data were collected from 12 clinical sites, which included 19 hospitals across the United States. This secondary analysis was conducted in February to July 2021.

**Exposures:**

Using *International Classification of Diseases, Ninth Revision*, codes and a validated algorithm to define disability, participants were classified as having physical, intellectual, sensory, or any disability, and compared with women with no documented disability.

**Main Outcomes and Measures:**

The relative risk (RR) of 23 obstetric interventions and adverse maternal outcomes, including SMM and mortality, was evaluated.

**Results:**

Of the 223 385 women in the study, 9206 (4.1%) were Asian or Pacific Islander, 50 235 (22.5%) were Black, 39 039 (17.5%) were Hispanic, and 110 443 (49.4%) were White, with a mean (SD) age of 27.6 (6.2) years. There were 2074 (0.9%) women with disability and 221 311 (99.1%) without. Among women with disabilities, 1733 (83.5%) were physical, 91 (4.4%) were intellectual, and 250 (12.1%) were sensory. Compared with women with no disability, women with disabilities had higher risk of gestational diabetes, placenta previa, premature rupture of membranes, preterm premature rupture of membranes, and postpartum fever as well as maternal death (adjusted relative risk [aRR], 11.19; 95% CI, 2.40-52.19) and individual SMMs: severe preeclampsia/eclampsia (aRR, 2.15; 95% CI, 1.80-2.56), hemorrhage (aRR, 1.27; 95% CI, 1.09-1.49), and fever (aRR, 1.32; 95% CI, 1.03-1.67), with the highest risk observed for thromboembolism (aRR, 6.08; 95% CI, 4.03-9.16), cardiovascular events (aRR, 4.02; 95% CI, 2.87-5.63), and infection (aRR, 2.69; 95% CI, 1.97-3.67). Women with any disability also had higher risk of interventions, including oxytocin augmentation, operative vaginal delivery, and cesarean delivery (aRR, 1.33; 95% CI, 1.25-1.42), with the cesarean indication less likely to be medically indicated (aRR, 0.79; 95% CI, 0.70-0.89). Risk of adverse outcomes and interventions remained consistent across disability categories.

**Conclusions and Relevance:**

In this study, women with physical, intellectual, and sensory disability during pregnancy were at higher risk of adverse outcomes, including a broad range of SMM and maternal mortality.

## Key Points

**Question:**

Is maternal disability associated with adverse maternal outcomes, including severe maternal morbidity and mortality?

**Findings:**

In this cohort study of 223 385 women, women with disabilities had higher risk of maternal mortality and all severe maternal morbidities, including severe preeclampsia/eclampsia, hemorrhage, fever, thromboembolism, cardiovascular events, and infection. They also had higher risk of obstetric intervention, with cesarean delivery less likely to be solely clinically indicated.

**Meaning:**

In this study, women with disabilities were at increased risk of a broad spectrum of adverse maternal outcomes and obstetric intervention that may increase risk for mortality.

## Teaser

This cohort study evaluates risk of a range of obstetric interventions and adverse maternal outcomes, including severe maternal morbidities and mortality, among women with and without disabilities.

## Introduction

Maternal mortality is a preventable and pressing public health issue, particularly among traditionally marginalized populations.^1^ Women with disabilities have a higher risk of pregnancy complications, including preterm birth, hypertensive disorders during pregnancy, gestational diabetes, and cesarean delivery.^2^ However, little is known about the risk of maternal mortality or severe maternal morbidities (SMM), such as maternal hemorrhage, infection, cardiovascular events, or thromboembolism, in women with disabilities. Given the high rate of maternal mortality in the United States and an increasing rate of pregnancy among women with disabilities,^3^ a population with significant environmental and behavioral risk factors,^4,5,6^ it is critical to evaluate whether women with disabilities are at higher risk than women without disability for other adverse outcomes, including SMM and mortality. To our knowledge, only 1 study^7^ has previously explored SMM among women with disabilities, although it did not evaluate risk of other adverse birth outcomes or explore individual components of SMM that may drive the composite risk. Additionally, although it is known that women with disabilities are more likely to have a cesarean delivery, reasons for increased risk have been understudied.^8^

One reason for this dearth of knowledge could be previous studies’ reliance on hospital administrative data, which are limited in scope and may not adequately characterize SMM that substantially contribute to maternal mortality.^9^ Additionally, most studies used data from outside the United States or from single states within the United States, limiting generalizability of findings. Only 3 studies that we are aware of used data from multiple US states, and these studies were limited by their relatively small number of women classified as having a disability^10,11^ or other methodological challenges, such as incomplete reporting on covariate information that could explain observed associations.^12^ In this study, we analyzed data from a large, multisite retrospective cohort study to conduct a comprehensive analysis of obstetric interventions and adverse maternal outcomes, including SMM and mortality, among women with and without documented disability.

## Methods

We used data from the Consortium on Safe Labor (CSL), a cohort of deliveries from 12 US clinical centers (19 hospitals) between January 2002 and January 2008. Details of this retrospective cohort have been described in detail,^13^ but briefly, researchers extracted data from electronic medical records for both women and their neonates. *International Classification of Diseases, Ninth Revision, Clinical Modification* (*ICD-9-CM*) codes were also collected and linked to deliveries. Extracted maternal information included demographic characteristics, medical and reproductive history, obstetric interventions, and postpartum complications. Neonatal information included gestational age, discharge summary, and medical conditions. A validation study of the electronic medical records with manual medical record review of key variables demonstrated high accuracy with greater than 91% concordance for all variables and greater than 95% for most.^13^ The procedures of the CSL and waivers for participants’ informed consent were approved by the institutional review boards for all participating institutions. This secondary analysis was conducted from February to July 2021. Of the 228 438 deliveries included in the CSL, we excluded multiples (n = 5053), using data from 223 385 singleton deliveries. This report followed the Strengthening the Reporting of Observational Studies in Epidemiology (STROBE) reporting guidelines.

### Adverse Maternal and Obstetric Outcomes

Outcomes were extracted from the electronic medical record and supplemented with *ICD-9* codes with definitions consistent with previous studies in the CSL.^14,15^ Maternal and obstetric outcomes included pregnancy-related hypertensive disorders (gestational hypertension, mild preeclampsia, and combined severe preeclampsia or eclampsia), gestational diabetes, placental abruption, placenta previa, premature rupture of membranes (PROM), preterm (<37 weeks) PROM (PPROM), prelabor (planned) and intrapartum cesarean delivery, induction of labor, oxytocin augmentation, operative vaginal delivery, chorioamnionitis, hemorrhage, blood transfusion, maternal thromboembolism, postpartum fever, infection (a composite of endometritis, major puerperal infection, wound infection or separation, or sepsis/septicemia), cardiovascular events during labor and delivery, maternal cardiomyopathy, and maternal death. *ICD-9* codes used to identify outcomes not directly extracted from the medical record are presented in eTable 1 in the Supplement. We also evaluated individual indications for cesarean birth and hierarchical indication groups: clinically indicated, mixed indications, and truly elective as done previously.^13^

To assess overall risk of adverse maternal outcomes in women with disabilities, we created 3 composite measures: (1) comprised of all pregnancy-related complications (pregnancy-related hypertensive diseases, gestational diabetes, placental abruption, placenta previa, PROM, PPROM); (2) all labor, delivery, and postpartum complications (chorioamnionitis, hemorrhage, blood transfusion, thromboembolism, postpartum fever, infection, cardiovascular events, cardiomyopathy, and maternal death); and (3) only SMM, including severe preeclampsia/eclampsia, hemorrhage, thromboembolism, fever, infection, cardiomyopathy, and cardiovascular events during labor and delivery. The morbidities selected for the SMM composite have previously been identified as the leading causes of maternal mortality in the United States^16^ and are similar to those included in the US Centers for Disease Control and Prevention definition of SMM.^17^

### Disability Status

Maternal disability was classified according to *ICD-9 *codes recorded in the discharge summary (eTable 2 in the Supplement). Disability definitions were based on an algorithm developed by Darney et al,^18^ which built on previous work to provide a list of codes likely to be associated with a wide range of functional impairment across disability categories and consists of 3 categories: physical, intellectual and developmental, and sensory (both hearing and visual) disability. Of note, some conditions, such as cerebral palsy, that might be associated with both physical and intellectual/developmental disabilities were only coded as 1 type of disability (physical), per the algorithm definition. Though some women had multiple *ICD-9* codes within disability categories, none had codes that would classify them in multiple categories; thus, disability categories were mutually exclusive. We also created a binary variable for disability, pooling women across categories as any disability. Women without an established *ICD-9* code for disability were considered to have no documented disability (referred to throughout as women without disabilities) and were used as the reference group for all analyses.

### Covariates

Covariates were selected based on established associations between disability and maternal, obstetric, and neonatal outcomes. Specifically, we adjusted all analyses for maternal age (years), parity (nulliparous or parous), marital status (married or not), insurance type (private, public or self-pay, other or unknown), prepregnancy body mass index (BMI; calculated as weight in kilograms divided by height in meters squared), smoking (yes or no) and alcohol use (yes or no) during pregnancy, clinical site, and history of any chronic disease (asthma, depression, anxiety, HIV, hypertension, pregestational diabetes, or kidney, heart, or thyroid disease). We additionally adjusted for race, as defined in the medical record (Asian or Pacific Islander, Hispanic, non-Hispanic Black, non-Hispanic White, or other or unknown) because it may serve as a proxy for unmeasured socioeconomic factors. We multiply imputed missing covariate data (BMI for 74 994 women; marital status for 7215 women) using chained equations with 20 imputations.

### Statistical Analysis

We calculated descriptive statistics to compare maternal and obstetric characteristics between women with and without disabilities, using *t* tests for continuous variables and χ^2^ tests for categorical variables. All statistical tests were 2-sided with a priori significance set at *P* = .05.

For primary analyses, to estimate the relative risk (RR) of obstetric interventions and adverse maternal outcomes, we fit Poisson regression models with robust standard errors^19^ for each individual outcome, comparing each disability category (any disability, physical, intellectual, sensory) to women with no disability. Because 19 215 of 223 385 women (8.6%) had multiple pregnancies in the CSL, we used generalized estimating equations to account for nonindependence. In secondary analyses, we crafted similar models for composite maternal outcomes, as well as indications for cesarean delivery and hierarchical indication groups for cesarean delivery (clinically indicated, mixed indication, truly elective). Due to small cell sizes, we only compared individual cesarean delivery indications between women with any disability and those with no disability. All analyses were considered exploratory and performed using SAS version 9.4 (SAS Institute).

## Results

Of the 223 385 women in the study, 9206 (4.1%) were Asian or Pacific Islander, 50 235 (22.5%) were Black, 39 039 (17.5%) were Hispanic, and 110 443 (49.4%) were White, with a mean (SD) age of 27.6 (6.2) years. There were 2074 (0.9%) women with disability and 221 311 without. Among women with disabilities, 1733 (83.5%) were physical, 91 (4.4%) were intellectual, and 250 (12.1%) were sensory. Compared with women with no recorded disability, women with disabilities were more likely to be non-Hispanic White, unmarried, use public insurance or self-pay, have a higher pre-pregnancy BMI, smoke, and have a history of chronic disease (Table 1).

**Table 1.** Medical and Demographic Characteristics by Disability Status, Consortium on Safe Labor

Characteristic	Participants, No. (%)					
	Total (N = 223 385)	Disability				
		None (n = 221 311)	Any (n = 2074)	Physical (n = 1733)	Intellectual (n = 91)	Sensory (n = 250)
Maternal age, mean (SD), y^a^	27.6 (6.2)	27.6 (6.2)	28.8 (6.4)	29.0 (6.3)	25.2 (7.1)	29.0 (6.5)
Maternal race^a^						
Asian/Pacific Islander	9206 (4.1)	9164 (4.1)	42 (2.0)	34 (2.0)	1 (1.1)	7 (2.8)
Black non-Hispanic	50 235 (22.5)	49 841 (22.5)	394 (19.0)	309 (17.8)	25 (27.5)	60 (24.0)
Hispanic	39 039 (17.5)	38 811 (17.5)	228 (11.0)	178 (10.3)	13 (14.3)	37 (14.8)
White non-Hispanic	110 443 (49.4)	109 149 (49.3)	1294 (62.4)	1124 (64.9)	43 (49.4)	127 (50.8)
Other or multiracial^b^	14 461 (6.5)	14 345 (6.5)	116 (5.6)	88 (5.1)	9 (9.9)	19 (7.6)
Parity						
Nulliparous	89 030 (39.9)	88 178 (39.8)	852 (41.1)	700 (40.4)	51 (56.0)	101 (40.4)
Parous, ie, ≥1 child	134 355 (60.2)	133 133 (60.2)	1222 (58.9)	1033 (59.6)	40 (44.0)	149 (59.6)
Marital status^a^						
Married	131 175(58.7)	130 039 (58.8)	1136 (54.8)	981 (56.6)	25 (27.5)	130 (52.0)
Not married	84 995 (38.1)	84 136 (38.0)	859 (41.4)	690 (39.8)	63 (69.2)	106 (42.4)
Unknown	7215 (3.2)	7136 (3.2)	79 (3.8)	62 (3.6)	3 (3.3)	14 (5.6)
Insurance type^a^						
Private	124 913 (55.9)	123 795 (55.9)	1118 (53.9)	990 (57.1)	21 (23.1)	107 (42.8)
Public or self-pay	74 810 (33.5)	74 004 (33.4)	806 (38.9)	620 (35.8)	58 (63.7)	128 (51.2)
Other or unknown	23 662 (10.6)	23 512 (10.6)	150 (7.2)	123 (7.1)	12 (13.2)	15 (6.0)
Pre-pregnancy BMI, mean (SD)	25.4 (6.2)	25.4 (6.2)	26.3 (6.9)	26.4 (7.0)	25.6 (6.0)	25.9 (6.7)
Prior cesarean delivery	30 641 (13.7)	30 284 (13.7)	357 (17.2)	301 (17.4)	9 (9.9)	47 (18.8)
Maternal history of chronic disease^a^^,^^c^	39 795 (17.8)	39 054 (17.7)	741 (35.7)	603 (34.8)	35 (38.5)	103 (41.2)
Smoking during pregnancy^a^	14 930 (6.7)	14 700 (6.6)	230 (11.1)	196 (11.3)	11 (12.1)	23 (9.2)
Alcohol use during pregnancy	4090 (1.8)	4051 (1.8)	39 (1.9)	31 (1.8)	3 (3.3)	5 (2.0)
Birth weight, mean (SD), g	3244 (607)	3246 (605)	3066 (718)	3095 (693)	2798 (843)	2965 (808)
GA at delivery, mean (SD), wk^a^	38.6 (2.4)	38.6 (2.3)	37.6 (3.0)	37.8 (2.9)	36.7 (3.8)	37.0 (3.7)

Abbreviations: BMI, body mass index (calculated as weight in kilograms divided by height in meters squared); GA, gestational age.

^a^ Significant difference (*P* < .05) between women with disability and no disability.

^b^ Other/Unknown race category includes any race reported in the medical record other than the listed categories, which includes multiracial and unknown race/ethnicity.

^c^ Chronic disease includes history of asthma, depression or anxiety, HIV, hypertension, pregestational diabetes, kidney disease, heart disease, or thyroid disease.

### Pregnancy Complications

After adjusting for maternal demographic and health history, compared with women without disabilities, women with disabilities had significantly higher risk of almost all adverse maternal outcomes, including any pregnancy-related hypertensive disorder, gestational diabetes, placenta previa, PROM, and PPROM (Table 2). The risk of gestational hypertension was approximately the same for women with disabilities as those without, but the risk of mild preeclampsia was 48% higher (adjusted RR [aRR], 1.48; 95% CI, 1.20-1.81) for women with disabilities, and the risk of severe preeclampsia/eclampsia was doubled (aRR, 2.15; 95% CI, 1.80-2.56) for women with disabilities. The magnitudes of association were consistent across disability types, although with less precision for some rarer outcomes. Compared with women without disabilities, the composite risk of any pregnancy complication was elevated 27% (aRR, 1.27; 95% CI, 1.16-1.38) for women with physical disabilities, 49% (aRR, 1.49; 95% CI, 1.06-2.10) for women with intellectual disabilities, and 53% (aRR, 1.53; 95% CI, 1.26-1.86) for women with sensory disabilities.

**Table 2.** Risk of Maternal and Obstetric Outcomes Among 223 385 Women Included in the Consortium on Safe Labor, Comparing Categories of Disability^a^

Outcome	Disability									
	None		Any		Physical		Intellectual		Sensory	
	No. (%)	RR (95% CI)	No. (%)	RR (95% CI)	No. (%)	RR (95% CI)	No. (%)	RR (95% CI)	No. (%)	RR (95% CI)
Pregnancy-related hypertensive disorders										
Any	18 469 (8.4)	1 [Reference]	286 (13.8)	1.50 (1.34-1.69)	208 (12.0)	1.37 (1.19-1.57)	16 (17.6)	1.67 (1.02-2.72)	62 (24.8)	2.43 (1.89-3.13)
Gestational hypertension	6017 (2.7)	1 [Reference]	61 (2.9)	0.93 (0.72-1.20)	49 (2.8)	0.91 (0.69-1.21)	2 (2.2)	0.61 (0.15-2.44)	10 (4.0)	1.16 (0.62-2.16)
Mild preeclampsia	6979 (3.2)	1 [Reference]	94 (4.5)	1.48 (1.20-1.81)	74 (4.3)	1.41 (1.12-1.77)	8 (8.8)	2.55 (1.27-5.11)	12 (4.8)	1.50 (0.85-2.65)
Severe preeclampsia/eclampsia	5473 (2.5)	1 [Reference]	131 (6.3)	2.15 (1.80-2.56)	85 (4.9)	1.76 (1.42-2.19)	6 (6.6)	1.81 (0.81-4.03)	40 (16.0)	4.29 (3.13-5.89)
Gestational diabetes	11 178 (5.1)	1 [Reference]	162 (7.8)	1.25 (1.07-1.46)	141 (8.1)	1.30 (1.10-1.53)	6 (6.6)	1.42 (0.64-3.16)	15 (6.0)	0.91 (0.55-1.51)
Placental abruption	3583 (1.6)	1 [Reference]	36 (1.7)	1.12 (0.81-1.56)	30 (1.7)	1.13 (0.78-1.62)	1 (1.1)	0.71 (0.10-5.06)	5 (2.0)	1.23 (0.51-2.96)
Placenta previa	1559 (0.7)	1 [Reference]	25 (1.2)	1.52 (1.02-2.26)	24 (1.4)	1.75 (1.16-2.63)	0	0	1 (0.4)	0.46 (0.07-3.30)
PROM	15 402 (7.0)	1 [Reference]	186 (9.0)	1.16 (1.02-1.34)	152 (8.8)	1.14 (0.97-1.33)	14 (15.4)	1.99 (1.18-3.36)	20 (8.0)	1.05 (0.68-1.63)
Preterm PROM	5020 (2.3)	1 [Reference]	91 (4.4)	1.55 (1.26-1.91)	74 (4.3)	1.52 (1.20-1.91)	8 (8.8)	3.00 (1.50-6.01)	9 (3.6)	1.24 (0.64-2.38)
Cesarean delivery										
Any	61 653 (27.9)	1 [Reference]	905 (43.6)	1.34 (1.26-1.43)	741 (42.8)	1.33 (1.23-1.43)	42 (46.2)	1.49 (1.10-2.01)	122 (48.8)	1.39 (1.17-1.67)
Prelabor cesarean delivery	25 422 (11.5)	1 [Reference]	434 (20.9)	1.46 (1.33-1.61)	359 (20.7)	1.47 (1.32-1.63)	17 (18.7)	1.54 (0.95-2.47)	58 (23.2)	1.42 (1.10-1.85)
Intrapartum cesarean delivery	36 231 (16.4)	1 [Reference]	471 (22.7)	1.25 (1.14-1.37)	382 (22.0)	1.22 (1.10-1.35)	25 (27.5)	1.46 (0.98-2.15)	64 (25.6)	1.36 (1.07-1.74)
Induction of labor	76 434 (34.5)	1 [Reference]	715 (34.5)	0.99 (0.92-1.07)	610 (35.2)	1.02 (0.94-1.10)	33 (356.3)	0.95 (0.68-1.34)	72 (28.8)	0.80 (0.64-1.01)
Oxytocin augmentation	64 155 (29.0)	1 [Reference]	744 (35.9)	1.08 (1.01-1.16)	620 (35.8)	1.10 (1.01-1.19)	37 (40.7)	1.10 (0.80-1.52)	87 (34.8)	0.93 (0.76-1.15)
Operative vaginal delivery	10 426 (6.5)	1 [Reference]	99 (8.5)	1.33 (1.27-1.39)	83 (8.4)	1.31 (1.25-1.38)	6 (12.2)	1.48 (1.21-1.82)	10 (7.8)	1.37 (1.21-1.55)
Chorioamnionitis	6863 (3.1)	1 [Reference]	55 (2.7)	1.10 (0.85-1.45)	41 (2.4)	1.02 (0.75-1.39)	5 (5.5)	1.62 (0.67-3.89)	9 (3.6)	1.45 (0.75-2.79)
Hemorrhage	14 565 (6.6)	1 [Reference]	147 (7.1)	1.27 (1.08-1.49)	115 (6.6)	1.19 (0.99-1.43)	6 (6.6)	1.23 (0.55-2.74)	26 (10.4)	1.80 (1.22-2.64)
Blood transfusion^b^	5168 (3.8)	1 [Reference]	59 (4.2)	1.64 (1.27-2.12)	45 (3.8)	1.52 (1.13-2.04)	2 (3.4)	1.44 (0.36-5.77)	12 (6.9)	2.42 (1.38-4.27)
Maternal thromboembolism	397 (0.18)	1 [Reference]	26 (1.3)	6.08 (4.03-9.16)	20 (1.2)	5.64 (3.54-8.96)	0	0	6 (2.4)	10.65 (4.73-24.04)
Postpartum fever	5993 (2.7)	1 [Reference]	68 (3.3)	1.32 (1.03-1.67)	58 (3.4)	1.40 (1.09-1.80)	4 (4.4)	1.45 (0.55-3.88)	6 (2.4)	0.81 (0.36-1.80)
Infection										
Any	2071 (0.9)	1 [Reference]	42 (2.0)	2.69 (1.97-3.67)	26 (1.5)	2.11 (1.42-3.13)	1 (1.1)	1.01 (0.14-7.14)	15 (6.0)	6.22 (3.74-10.37)
Endometritis	571 (0.5)	1 [Reference]	5 (0.5)	1.35 (0.56-3.28)	3 (0.3)	NA^c^	0	0	2 (1.4)	NA^c^
Major puerperal infection	1124 (0.5)	1 [Reference]	20 (1.0)	1.71 (1.08-2.70)	12 (0.69)	1.30 (0.72-2.36)	0	0	8 (3.2)	4.50 (2.24-9.03)
Wound infection or separation	738 (0.3)	1 [Reference]	13 (0.6)	NA^c^	12 (0.7)	NA^c^	0	0	1 (0.4)	NA^c^
Sepsis or septicemia	61 (0.03)	1 [Reference]	17 (0.8)	23.0 (13.0-40.6)	3 (0.2)	4.66 (1.43-15.19)	1 (1.1)	23.2 (3.17-168.9)	13 (5.2)	143.2 (76.3-268.6)
Cardiovascular event during labor and delivery	680 (0.3)	1 [Reference]	38 (1.8)	4.02 (2.87-5.63)	29 (1.7)	3.90 (2.65-5.73)	91 (1.1)	2.07 (0.29-14.77)	8 (3.2)	5.18 (2.57-10.43)
Maternal cardiomyopathy	63 (0.03)	1 [Reference]	3 (0.14)	NA^c^	3 (0.17)	NA^c^	0	0	0	0
Maternal death	18 (0.01)	1 [Reference]	2 (0.09)	11.19 (2.40-52.19)	0	0	2 (2.1)	NA^c^	0	0

Abbreviations: NA, not applicable; PROM, premature rupture of membranes; RR, relative risk.

^a^ All analyses adjusted for maternal age, race, parity, marital status, insurance type, pre-pregnancy BMI, smoking and alcohol use during pregnancy, clinical site, and history of any chronic disease.

^b^ Due to incomplete reporting, blood transfusion available at only 6 sites.

^c^ Models do not converge to provide estimates.

### Obstetric Interventions

Women with disabilities also had higher risk of obstetric intervention, including oxytocin augmentation during labor (aRR, 1.08; 95% CI, 1.01-1.16), operative vaginal delivery (aRR, 1.33; 95% CI, 1.27-1.39), and both prelabor (planned) and intrapartum cesarean delivery, but not labor induction (women with disabilities, 715 of 2074 [34.5%]; women without disabilities, 76 434 of 221 311 [34.5%]). These patterns remained mostly consistent across disability type, although there was loss of precision for some of the associations, as the number of cases was low in some categories.

Given that women with disabilities had higher risk of cesarean delivery (aRR, 1.34; 95% CI, 1.26-1.43), indications for cesarean delivery were examined. Compared with women without disability, women with disability had a decreased risk of cesarean delivery for failure to progress or cephalopelvic disproportion and increased risk of cesarean delivery for pregnancy-related hypertensive disease, fetal indication or anomaly, and indication labeled as other or unknown (aRR, 1.76; 95% CI, 1.50-2.07) (eTable 3 in the Supplement). When combining indications into hierarchical groups (Table 3), compared with women without a disability, women with any disability and those in each disability category were less likely to have a cesarean delivery that was solely clinically indicated (aRR, 0.79; 95% CI, 0.70-0.89), but more likely to have a cesarean delivery that was of mixed indication (aRR, 1.16; 95% CI, 1.06-1.28).

**Table 3.** Hierarchical Indications for Cesarean Delivery Among 61 653 Women Included in the Consortium on Safe Labor

Hierarchical indication	Disability									
	None		Any		Physical		Intellectual		Sensory	
	No. (%)	RR (95% CI)	No. (%)	RR (95% CI)	No. (%)	RR (95% CI)	No. (%)	RR (95% CI)	No. (%)	RR (95% CI)
Clinically indicated^a^	24 179 (39.4)	1 [Reference]	277 (30.7)	0.80 (0.71-0.90)	225 (30.5)	0.79 (0.69-0.90)	18 (42.9)	0.96 (0.60-1.52)	34 (27.9)	0.72 (0.52-1.01)
Mixed indication^b^	29 085 (47.4)	1 [Reference]	454 (50.3)	1.16 (1.06-1.28)	366 (49.6)	1.15 (1.04-1.28)	21 (50.0)	1.22 (0.80-1.87)	67 (54.9)	1.22 (0.96-1.56)
Truly elective^c^	8166 (13.3)	1 [Reference]	171 (19.0)	1.04 (0.89-1.21)	147 (19.9)	1.09 (0.92-1.28)	3 (7.1)	0.48 (0.15-1.48)	21 (17.2)	0.94 (0.61-1.45)

^a^ Clinically indicated includes the following indications for cesarean delivery: emergency, nonreassuring fetal heart trace, failure to progress/cephalopelvic disproportion, failed induction, failed forceps, failed vaginal delivery after cesarean, shoulder dystocia, history of shoulder dystocia, placenta previa, and placental abruption.

^b^ Mixed indications include previous uterine scar, breech presentation, macrosomia, fetal indication or anomaly, HIV, hypertensive disease, and other indication (other indications included anything that did not fit into any other listed category).

^c^ Elective indications include maternal request, multiparity, those who desired tubal ligation, advanced maternal age, diabetes, human papillomavirus, post term or post dates, remote from term, group B *Streptococcus*, polyhydramnios, fetal demise, and social or religious concern.

### Adverse Labor, Delivery, and Postpartum Outcomes

Women with disabilities had a higher risk of postpartum hemorrhage (aRR, 1.27; 95% CI, 1.08-1.49) and blood transfusion (aRR, 1.64; 95% CI, 1.27-2.12). They also had higher risk of all SMM (Table 2), including postpartum fever (aRR, 1.32; 95% CI, 1.03-1.67), maternal thromboembolism (aRR, 6.08; 95% CI, 4.03-9.16), cardiovascular events (aRR, 4.02; 95% CI, 2.87-5.63), and infection (aRR, 2.69; 95% CI, 1.97-3.67), the composite of which included particularly high risk of sepsis/septicemia (aRR, 23.04; 95% CI, 13.01-40.57). Although there were few maternal deaths, women with disabilities had higher mortality risk compared with women without disabilities (aRR, 11.19; 95% CI, 2.40-52.19). Heightened risk of individual morbidity outcomes varied but was generally consistent across disability categories. For example, women with sensory disabilities had 6.22 (95% CI, 3.74-10.37) times higher risk of infection than women with no recorded disability, while women with physical disabilities had 3.90 (95% CI, 2.65-5.73) times higher risk of cardiovascular events, and women with intellectual disabilities had 1.76 (95% CI, 1.42-2.19) times higher risk of severe preeclampsia/eclampsia. Maternal thromboembolism risk was markedly increased for both physical (aRR, 5.64; 95% CI, 3.54-8.96) and sensory disabilities (RR, 10.65; 95% CI, 4.73-24.04), with no cases in women with intellectual disability. The composite risk of any labor, delivery, or postpartum complication was 1.36 (95% CI, 1.21-1.53) for any disability, 1.33 (95% CI, 1.17-1.51) for physical disability, 1.14 (95% CI, 0.64-2.00) for intellectual disability, and 1.67 (95% CI, 1.25-2.22) for sensory disability. Women with any disability were at higher risk of any SMM (aRR, 1.59; 95% CI, 1.43-1.76), with the highest risk observed in women with sensory disability (aRR, 2.39; 95% CI, 1.89-3.01) (Figure).

**Figure.**
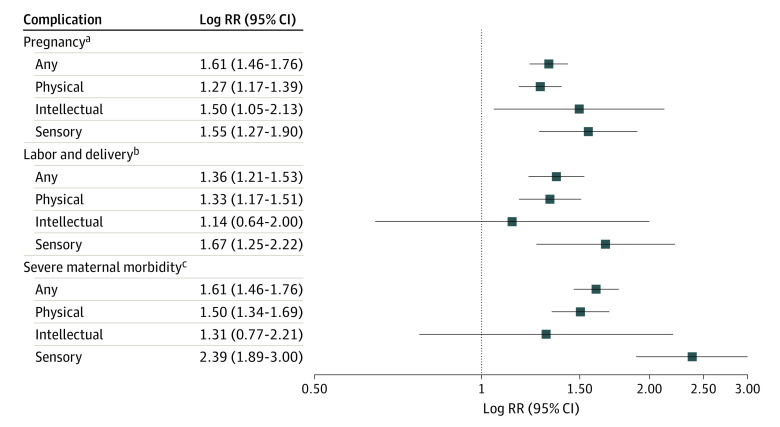
Risk of Experiencing Any Pregnancy or Labor, Delivery, or Postpartum Complication by Disability Status ^a^Maternal pregnancy composite includes pregnancy-related hypertensive diseases, gestational diabetes, placental abruption, placenta previa, premature rupture of membranes, and preterm premature rupture of membranes. ^b^Labor and delivery composite includes chorioamnionitis, hemorrhage, blood transfusion, thromboembolism, postpartum fever, infection, cardiovascular events, cardiomyopathy, and maternal death. ^c^Severe maternal morbidity composite includes severe preeclampsia/eclampsia, hemorrhage, thromboembolism, fever, endometritis, major puerperal infection, wound infection/separation, sepsis, cardiomyopathy, and cardiovascular event.

## Discussion

Women with disabilities had higher risk of almost all pregnancy complications, obstetric interventions, and adverse outcomes, including SMM and mortality. Heightened risk occurred in each of the 3 disability categories: physical, intellectual, and sensory. Furthermore, we observed increased risks of relatively rare outcomes on which other studies have not reported, including comprehensive evaluations of cardiovascular events, maternal infections, and maternal mortality. Despite the increased risk of pregnancy complications, including gestational diabetes and preeclampsia, there was not a corresponding increase in labor induction. However, there was an increased risk of prelabor (ie, planned) cesarean and intrapartum cesarean birth, suggesting a potential health care practitioner preference for this more invasive method of obstetric management vs induction.^20^ The indications for cesarean delivery for women with disability were more likely for softer indications compared with true clinical indications for women without disability, supporting a possible overuse of cesarean delivery among women with disability. Cesarean delivery is a noted risk factor for SMM and maternal mortality,^21^ which, combined with a potential lack of awareness of the high-risk pregnancy status among women with disability,^22,23^ may have facilitated the observed increases in SMM and mortality.

Our findings are novel in light of the small body of systematic research conducted on women with disabilities.^24^ Only 1 study^7^ of which we are aware previously explored SMM and mortality among women with disabilities, finding an increased risk of a composite of SMM and mortality among women with physical, intellectual, sensory, or multiple types of disability using Canadian administrative data. However, that article did not evaluate individual components of SMM, which may miss individual outcomes that substantially increase the risk of the composite. In the context of our findings, we observed that women with disabilities had elevated risk of all top SMM associated with mortality, ie, obstetrical hemorrhage, preeclampsia/eclampsia, venous thromboembolism, infections, and cardiovascular events. Understanding the risk of individual components of SMM may facilitate the planning of specific interventions among women with disabilities to reduce risk of SMM and maternal mortality.

Our findings of increased risk of pregnancy-related hypertensive disorders and cesarean delivery are consistent with a systematic review and meta-analysis, and increased risk of gestational diabetes has also been observed, although with inconsistent results.^2^ However, our study expands knowledge on how risk remains heightened or occasionally differs among women with different types of disability. Of the other studies exploring outcomes beyond pregnancy complications and cesarean birth, most are restricted to one type of disability,^10,11,25^ and those that explore multiple disabilities have only additionally evaluated postpartum hospital utilization.^12,26,27^ Specifically, to our knowledge, only 1 other study^28^ has examined thromboembolism and maternal mortality beyond descriptive statistics, but it was conducted among women with spinal conditions only, and no other study of which we are aware has evaluated blood transfusion, postpartum fever, multiple types of postpartum infection, or cardiovascular events while controlling for potential confounding factors. It is also interesting to note that relative to women with no recorded disability, women with all types of disability had higher risk of mild and severe preeclampsia and eclampsia, but not gestational hypertension, suggesting that these more severe hypertensive disorders are driving the risk of having any hypertensive disorder. This finding may be important to note for studies that evaluate a composite outcome of hypertensive disorders. Specifically, grouping gestational hypertension with preeclampsia and eclampsia may lead to an underestimation of the risk of severe hypertensive disorders in this population. Additionally, although the risk of cesarean delivery is consistently recognized to be higher in women with disabilities,^2^ we expanded on existing evidence describing indications,^8^ finding that cesarean delivery was less likely to be solely clinically indicated and observing a higher risk of cesarean delivery indications for hypertension, fetal indication, or other/unknown indication. In total, observing an increased risk of cesarean delivery, particularly for other/or unknown indications, but not induction of labor, may reflect a health care practitioner preference to avoid natural labor in women with disabilities, as described in other studies.^20,29^

Women with disabilities often have higher risk factors for poor maternal outcomes, including higher likelihood of living in poverty, smoking, substance use, and depression.^4,5^ They also face barriers to care, including financial barriers that could delay seeking care and physically accessing health care facilities.^6^ In addition to this lack of tangible support, women with mobility impairments report lack of clinical knowledge of their disability when seeking prenatal care.^30,31^ This lack of clinical knowledge has been supported by physician reports describing lack of education at every level of training to support pregnant women with disabilities,^22^ including a study^23^ that found clinical residents were uncomfortable managing pregnancies in women with disabilities. Women with disabilities also report negative reactions toward their pregnancy, which extends to health care practitioners and may affect the quality of care provided and lead to refusal of care for women with disabilities.^32,33^ These types of attitudinal barriers for women with disabilities were noted by the American College of Obstetricians and Gynecologists in 2005.^34^ In combination, lack of training and negative attitude lead to an informational barrier with little communication between the obstetrician and disability practitioner,^6^ the consequence of which is fragmented care that can cause problems during the prenatal period and labor and delivery. This combination of barriers may be exemplified by our finding that women with disability had higher risk of preeclampsia/eclampsia but not gestational hypertension. Perhaps a delay in care for any of the above reasons resulted in more progression of hypertension to preeclampsia when the patient presented for care. Our findings may be a direct reflection of the challenges women with all types of disabilities face when accessing and receiving care, which is likely compounded by poorer preconception health.^35^

A major strength of this study is the availability of comprehensive medical record information from 19 hospitals across the United States. Our outcomes were not limited to reports in claims or administrative records, which allowed us to evaluate risk of many outcomes not previously explored. Additionally, we had a large enough sample size to examine individual SMM among a small subset of our cohort to make statistically valid conclusions. We also had data on indications for cesarean delivery, which has, to our knowledge, not been previously evaluated but is an important contribution considering the independent risk of SMM and mortality associated with cesarean delivery.

### Limitations

This study has limitations, including the inability to evaluate actual functional limitation associated with disabilities documented by *ICD-9* codes or issues with access to antenatal care. However, given the large magnitude of risk observed for some outcomes, this functional limitation may be less relevant than the diagnosis, which may indicate some underlying pathology that increases risk of adverse outcomes. Another limitation of *ICD-9* codes is that only conditions that are actively addressed in a health care encounter are coded. Considering that we may only be capturing severe forms of disability that would be documented in the delivery record and that our disability-defining algorithm classified each diagnostic code into only one category of disability, this limitation may explain why we did not observe any women with multiple categories of disability. However, we do not believe this would change the interpretation of our findings, considering the consistency of magnitude and direction of estimates across disability categories. In the recent article by Brown et al^7^ on SMM in Canada, their findings for women with multiple types of disabilities were consistent with those of women with only one type of disability. For the same reasons, we may have inadvertently excluded some women with an undocumented disability that impairs function. We expect these examples of misclassification would be nondifferential across our sample and may have only attenuated our findings. Given the small number of women in the intellectual and sensory disability categories, we advise caution in interpreting results for outcomes with a small number of cases, even when statistically significant. Although our data are from 2002 to 2008, we believe our results are generalizable to a more modern cohort given that current cesarean delivery rates are the same as those at the mid-point of the CSL in 2004^36,37^ and recent studies have found similar increased risk of cesarean among women with disabilities.^8^

## Conclusions

In this study, women with physical, intellectual, and sensory disabilities had elevated risk for nearly all pregnancy, labor, delivery, and immediate postpartum outcomes, including maternal death and SMM that are the top contributors to maternal mortality. Increased risk may be the result of a combination of independent risk factors, including the higher rate of obstetric intervention via cesarean delivery, underrecognition of women with disabilities as a population with higher risk pregnancies, and lack of health care practitioner knowledge or comfort in managing pregnancies among women with disabilities. Interventions to reduce rates of SMM, maternal mortality, and other pregnancy complications in this population are need. Systemic implementation of evidence-based obstetric practice and care processes have been shown to reduce SMM and maternal mortality.^38^ Furthermore, changes in the medical education system and other strategies are needed to help health care practitioners be more comfortable managing care for reproductive-age women with disabilities before and during pregnancy. Future work should evaluate whether changes to the medical education system would improve outcomes.^22^

## Notes

Supplement. **eTable 1.** *International Classification of Diseases, Ninth Revision *(*ICD-9*) Codes Used to Define Maternal Thromboembolism and Cardiovascular Events During Labor and Delivery **eTable 2.** *International Classification of Diseases, Ninth Revision *(*ICD-9*) Codes Used to Define Disability **eTable 3.** Individual Indications for Cesarean Delivery for 61 653 Women Included in the Consortium on Safe Labor
