# Supplementary material for: Risk of Adverse Maternal Outcomes in Pregnant Women With Disabilities
Source: JAMA Netw Open. 2021 Dec 15;4(12):e2138414. doi: 10.1001/jamanetworkopen.2021.38414 (PMC8674748; doi:10.1001/jamanetworkopen.2021.38414)
Supplement: Supplement. — eTable 1. International Classification of Diseases, Ninth Revision (ICD-9) Codes Used to Define Maternal Thromboembolism and Cardiovascular Events During Labor and Delivery eTable 2. International Classification of Diseases, Ninth Revision (ICD-9) Codes Used to Define Disability eTable 3. Individual Indications for Cesarean Delivery for 61 653 Women Included in the Consortium on Safe Labor [file jamanetwopen-e2138414-s001.pdf]

Supplemental Online Content

Gleason JL, Grewal J, Chen Z, Cernich AN, Grantz KL. Risk of adverse maternal outcomes in pregnant women with disabilities. *JAMA Netw Open.* 2021;4(12):e2138414. doi:10.1001/jamanetworkopen.2021.38414

eTable 1. *International Classification of Diseases, Ninth Revision* (*ICD-9*) Codes Used to Define Maternal Thromboembolism and Cardiovascular Events During Labor and Delivery

eTable 2. *International Classification of Diseases, Ninth Revision* (*ICD-9*) Codes Used to Define Disability

eTable 3. Individual Indications for Cesarean Delivery for 61 653 Women Included in the Consortium on Safe Labor

This supplemental material has been provided by the authors to give readers additional information about their work.

© 2021 Gleason JL et al. *JAMA Network Open.*

eTable 1. *International Classification of Diseases, Ninth Revision* (*ICD-9*) Codes Used to Define Maternal Thromboembolism and Cardiovascular Events During Labor and Delivery

Condition for outcome classification	ICD-9 codes
**Maternal Thromboembolism**	
Other pulmonary embolism and infarction	415.19
Other venous embolism and thrombosis	453.x
Superficial thromboplebitis complicating pregnancy and the puerperium	671.21-24
Deep phlebothrombosis, antepartum, delivered, with or without mention of antepartum condition	671.31
Deep phlebothrombosis, antepartum, antepartum condition or complication	671.33
Deep phlebothrombosis, postpartum	671.42
Other phlebitis and thrombosis complicating pregnancy and the puerperium	671.51-54
Obstetrical air embolism, delivered, with or without mention of antepartum condition	673.01
Amniotic fluid embolism	673.11-12
Obstetrical blood-clot embolism	673.21-24
Obstetric pyemic and septic embolism	673.33
Other obstetric pulmonary embolism	673.8
Cerebrovascular disorders in the puerperium	674.01-04
**Cardiovascular Events during labor and delivery**	
Ischemic heart disease	
Acute myocardial infarction	410
Other acute and subacute forms of ischemic heart disease	411
Angina pectoris	413
Certain sequlae of myocardial infarction, not elsewhere classified	429.7
Stroke	
Subarachnoid hemorrhage	430
Intracerebral hemorrhage	431
Other and unspecified intracranial hemorrhage	432
Occlusion and stenosis of precerebral arteries	433
Occlusion of cerebral arteries	434
Transient cerebral ischemia	435
Acute, but ill-defined cerebrovascular disease	436
Cerebrovascular disorder in the puerperium	674
Central Nervous system complications - Complications of the administration of anesthetic or other sedation in labor and delivery	668.2
Other nervous system complications - Complications affecting specified body systems, not elsewhere classified	997.09

© 2021 Gleason JL et al. *JAMA Network Open.*

Heart failure	428
Cardiac arrest/failure	
Cardiac arrest	427.5
Complications of the administration of anesthetic or other sedation in labor and delivery - Cardiac complications	668.1
Other complications of obstetrical surgery and procedures	669.4
Complications affecting specified body systems, not elsewhere classified - Cardiac complications	997.1
Unspecified cardiovascular events	
Other cardiovascular diseases with postpartum complication	648.62 and 648.64

© 2021 Gleason JL et al. *JAMA Network Open.*

eTable 2. *International Classification of Diseases, Ninth Revision* (*ICD-9*) Codes Used to Define Disability

Condition for exposure classification	ICD-9 codes
**Physical disabilities**	
Multiple sclerosis	340
Cerebral palsy	343.x
Rheumatoid arthritis	714.x, 725.x
Ankylosing spondylitis	720
Spina bifida	741.x
Paralysis/hemiplegia	344.x, 342.x
Spinal injuries	806.x, 905.1, 907.x, 952.x, 953.x
Stroke – late effects	438.x
Polio (w/ paralysis, spinal, +late effects)	045.1x, 045.0x, 045.9x, 138.x
Myasthenia gravis	358.x
Muscular & myotonic dystrophies	359.x
Chondrodystrophy	756.4
Miscellaneous Physical disabilities	
Wheelchair dependence	V46.3
Dependence on other enabling machine	V46.8
Nervous System Disabilities	
Hereditary and Degenerative Diseases of the CNS	
Neurofibromatosis (von Recklinghausen's Disease)	237.7
Cerebral ataxia	331.89
Parkinson's Disease	332.0-332.1
Other extrapyramidal disease and abnormal movement disorders	333
Spinocerebellar disease	334
Spinal muscular atrophy	335.1
Motor neuron disease	335.2
Other anterior horn cell diseases	335.8, 335.9
Other diseases of the spinal cord	336
Disorders of the autonomic nervous system	337
Other CNS disorders	
Other demyelinating diseases of CNS	341
Epilepsy	345
Nerve root and plexus disorders	353
Hereditary and idiopathic peripheral neuropathy	356
Inflammatory and toxic neuropathy	357
Diseases of Muscloskeletal and Connective Tissue	
Acromegaly and gigantism	253
Osteoarthrosis	715
Internal derangement of the knee	717
Spondylosis and allied disorders	721

Disc disorders	722
Other disorders of the cervical region	723
Disorders of muscle ligament and fascia	728
Osteochondropathies	732
Injuries	
Crushing injury of the lower limb	928
Other acquired limb deformity of unspecified limb	736.89
Amputation of upper limb	V49.6
Amputation of lower limb	V49.7
Congenital Anomalies	
Cystic fibrosis	277
Dwarfism, not elsewhere classified	259.4
Other congenital anomalies of nervous system	742
Larsen syndrome	755.8
Congenital reduction deformities of upper limb	755.2
Congenital reduction deformities of lower limb	755.3
Syndactyly	755.1
Acrocephalosyndactyly (Apert syndrome)	755.55
Club foot and other congenital valgus deformities of feet	754.51-754.7
Other congenital musculoskeletal anomalies	756
**Intellectual disability**	
Mild, other, or unspecified intellectual disabilities	317.x – 319.x
DiGeorge syndrome (22q11.2 deletion)	279.11
Pervasive developmental disorders (includes ASD)	299
Cerebral degenerations usually manifest in childhood	330
Chromosomal anomalies for which a developmental disability is typically present	758.0-758.3
Other conditions due to autosomal anomalies	758.5
Other conditions due to chromosomal anomalies	758.89
Conditions due to anomaly of unspecified chromosome	758.9
Tuberous sclerosis	759.5
Other harmatoses	759.6
Multiple congenital anomalies not otherwise specified	759.7
Other specified congenital anomalies	759.8
Alcohol affecting fetus or newborn via placenta or breast milk	760.71
**Sensory disabilities**	
Hearing Disabilities	
Hearing impairment/deafness	389.x
Congenital anomalies of ear causing impairment of hearing	744
Vision Disabilities	
Visual impairment/blindness	369.x, 950.x
Disorders of the globe	360
Other retinal disorders	362

© 2021 Gleason JL et al. *JAMA Network Open.*

Chorioretinal inflammation, scars and other disorders of choroid	363
Disorders of iris and ciliary body	364
Glaucoma	365
Cataract	366
Disorders of visual cortex	377.7

© 2021 Gleason JL et al. *JAMA Network Open.*

eTable 3. Individual Indications for Cesarean Delivery for 61 653 Women Included in the Consortium on Safe Labor

	No disability		Any disability	
**Indications for cesarean**	n (%)	RR (95% CI)	n (%)	RR (95% CI)
Previous uterine scar	20,344 (31.3)	1 [Reference]	291 (30.4)	0.95 (0.84-1.07)
Failure to progress/cephalopelvic disproportion	14,482 (22.3)	1 [Reference]	151 (15.8)	0.71 (0.60-0.83)
Malpresentation	8339 (12.8)	1 [Reference]	101 (10.6)	0.83 (0.67-1.03)
Nonreassuring fetal heart tracing	10,467 (16.1)	1 [Reference]	138 (14.4)	0.90 (0.76-1.07)
Elective	8376 (12.9)	1 [Reference]	174 (18.2)	1.04 (0.90-1.22)
Hypertensive disease	1684 (2.6)	1 [Reference]	44 (4.6)	1.60 (1.15-2.22)
Macrosomia	1293 (2.0)	1 [Reference]	27 (2.8)	1.18 (0.80-1.73)
Fetal indication or anomaly	1059 (1.6)	1 [Reference]	28 (2.9)	1.94 (1.31-2.87)
Failed induction	685 (1.1)	1 [Reference]	6 (0.6)	0.58 (0.24-1.41)
Placenta previa or vasa previa	715 (1.1)	1 [Reference]	10 (1.0)	0.98 (0.52-1.84)
Chorioamnionitis	395 (0.6)	1 [Reference]	7 (0.7)	0.98 (0.44-2.20)
HIV, active herpes simplex virus lesions	470 (0.7)	1 [Reference]	4 (0.4)	0.42 (0.13-1.29)
Placental abruption	447 (0.7)	1 [Reference]	5 (0.5)	0.59 (0.22-1.58)
Emergency	247 (0.4)	1 [Reference]	3 (0.3)	0.82 (0.26-2.59)
Failed trial of forceps or vacuum	113 (0.2)	1 [Reference]	4 (0.4)	1.96 (0.72-5.34)
History of shoulder dystocia	33 (0.1)	1 [Reference]	2 (0.2)	N/A[a]
Failed vaginal birth after cesarean	46 (0.1)	1 [Reference]	0 (0)	0
Shoulder dystocia	17 (0.03)	1 [Reference]	0 (0)	0
Other [b] or unknown	5882 (9.0)	1 [Reference]	159 (16.6)	1.85 (1.57-2.17)

[a] Models do not converge to provide estimates.
[b] Other indications included anything that did not fit into any other listed category.
Note: Indications may add up to more than 100% because more than one indication could have been listed.

© 2021 Gleason JL et al. *JAMA Network Open.*
